# MSA-Net: a multi-scale and adversarial learning network for segmenting bone metastases in low-resolution SPECT imaging

**DOI:** 10.1186/s40658-025-00785-w

**Published:** 2025-07-24

**Authors:** Yusheng Wu, Qiang Lin, Yang He, XianWu Zeng, Yongchun Cao, ZhengXing Man, Caihong Liu, Yusheng Hao, Zhengqi Cai, Jinshui Ji, Xiaodi Huang

**Affiliations:** 1 Key Laboratory of China’s Ethnic Languages and Information Technology of Ministry of Education, Northwest Minzu University, Lanzhou, Gansu China; 2 School of Mathematics and Computer Science, Northwest Minzu University, Lanzhou, Gansu China; 3 Gansu Provincial Engineering Research Center of Multimodal Artificial Intelligence, Northwest Minzu University, Lanzhou, Gansu China; 4 Department of Nuclear Medicine, Gansu Provincial Cancer Hospital, Lanzhou, Gansu China; 5 School of Computing, Mathematics and Engineering, Charles Sturt University, Albury, NSW Australia

**Keywords:** SPECT image, Bone metastasis, Multiscale segmentation, Adversarial learning

## Abstract

**Background:**

Single-photon emission computed tomography (SPECT) plays a crucial role in detecting bone metastases from lung cancer. However, its low spatial resolution and lesion similarity to benign structures present significant challenges for accurate segmentation, especially for lesions of varying sizes.

**Methods:**

We propose a deep learning-based segmentation framework that integrates conditional adversarial learning with a multi-scale feature extraction generator. The generator employs cascade dilated convolutions, multi-scale modules, and deep supervision, while the discriminator utilizes multi-scale L1 loss computed on image-mask pairs to guide segmentation learning.

**Results:**

The proposed model was evaluated on a dataset of 286 clinically annotated SPECT scintigrams. It achieved a Dice Similarity Coefficient (DSC) of 0.6671, precision of 0.7228, and recall of 0.6196 — outperforming both classical and recent adversarial segmentation models in multi-scale lesion detection, especially for small and clustered lesions.

**Conclusion:**

Our results demonstrate that the integration of multi-scale feature learning with adversarial supervision significantly improves the segmentation of bone metastasis in SPECT imaging. This approach shows potential for clinical decision support in the management of lung cancer.

## Introduction

Radionuclide bone scanning is a widely used method for detecting metastatic lesions in lung cancer patients. The use of single-photon nuclide drugs, such as ^99m^Tc-MDP, highlights metastatic areas in a patient’s bones by converting them into regions of high nuclide uptake in single-photon emission computed tomography (SPECT) imaging. This provides crucial information for physicians to stage the disease, make prognostic determinations, and more. However, due to the low resolution and specificity of SPECT images, metastatic lesions of lung cancer, which can vary widely in scale, are often easily confused with benign lesions. This makes it difficult to manually and accurately determine the distribution of metastatic lesions in lung cancer patients. This challenge underscores the need for an automated and robust segmentation method that can detect and delineate lesions of varying sizes, shapes, and visual characteristics in low-quality, noise-prone SPECT images.

Image segmentation is a crucial task in the field of computer vision, aiming to decompose digital images into semantically meaningful regions or objects. Medical image segmentation techniques can automatically outline and identify human tissue structures or lesion areas, thereby supporting efficient diagnosis by physicians. With the rise of deep learning methods [1], convolutional neural networks (CNNs) have become the mainstream method in image segmentation due to their ability to automatically extract representative features from images and their superior performance compared to traditional algorithms. Building on CNNs, the U-Net model, inspired by the Fully Convolutional Network (FCN) [2] and encoder-decoder model, has been proposed and successfully applied in the biomedical field, achieving excellent results on smaller medical image datasets [3]. Various models from the U-Net family have been developed for different types of medical images [4–6].

In recent years, adversarial learning techniques have advanced significantly in image generation [7], image segmentation [8], and style transfer [9] due to their ability to reveal high-dimensional data distributions and extract richer image features. Liu et al. [10] proposed an attention-enhanced Wasserstein Generative Adversarial Network (WGAN) for retinal vessel segmentation in fundus images. Their ASU-Net model integrates attention-enhanced convolutions and squeeze-and-excitation modules to improve global feature extraction. However, the network’s discriminator outputs only binary classifications, resulting in insufficient and unstable gradient feedback. Khan et al. [11] introduced the Multi-Resolution Context Network (MRC-Net) for retinal vessel segmentation, which learns contextual dependencies by extracting multi-scale features. Nonetheless, the use of cascaded 3 × 3, 5 × 5, and 7 × 7 convolutions increases computational demands and is limited by available resources. Innani et al. [12] proposed Efficient-GAN (EGAN), which incorporates squeeze-and-excitation modules in the generator’s encoder to enhance representational capacity and an Atrous Spatial Pyramid Pooling (ASPP) module in the bottleneck layer to improve multi-scale feature learning. However, this model does not enhance multi-scale learning in other layers, and its discriminator, which relies solely on predicted and ground truth segmentation labels, fails to fully leverage rich contextual information, such as texture and structure, limiting its discriminative power.

To address these limitations, we formulate the following core research questions: (1) Can adversarial learning be effectively used to enhance multi-scale lesion segmentation in noisy, low-resolution SPECT images? (2) Can multi-scale feature extraction modules and deep supervision improve sensitivity to small and clustered lesions? In response to these questions, we propose a novel segmentation framework that integrates conditional adversarial learning with a multi-scale generator and a deeply supervised loss scheme.

Targeting the characteristics of SPECT images, where lesion scales vary significantly, this paper proposes a segmentation model based on conditional adversarial networks for lung cancer bone metastasis lesions. The model demonstrates effective performance in segmenting multi-scale lesions and accurately identifying lesions at various locations.

The main contributions of this work are as follows:

- We propose an adversarial learning framework specifically tailored for SPECT image segmentation, which introduces a multi-scale L1 loss that provides richer supervision signals during training.
- We design a novel multi-scale feature extraction module (MSFE) that effectively captures lesion features at different scales, improving the model’s sensitivity to small, irregularly shaped lesions.
- We integrate deep supervision and image pyramid inputs into the generator to enhance hierarchical feature representation and improve segmentation consistency across multiple scales.
- We evaluate our method on a clinically validated SPECT dataset of 286 SPECT images, demonstrating that it significantly outperforms both classical and state-of-the-art adversarial segmentation models.

## Methods

### Dataset

#### Bone scan images

The data used in this experiment were obtained from the Department of Nuclear Medicine, Gansu Provincial Cancer Hospital, using the osteophilic ^99m^Tc-MDP drug to detect bone lesions, and the image acquisition was performed by a Siemens SPECT ECAM imaging device. The resolution of the SPECT images was 256 (pixels) × 1024 (pixels), and the pixel pitch was 2.26 mm. The acquisition time for each whole-body bone scan image is 10–15 min. Unlike natural images, which have a pixel range of 0–255, each SPECT image is a 256 × 1024 matrix of values consisting of 16-bit unsigned integers, where each value represents the radiation value of a radioactive element. A patient examined by bone scanning produces two images in the anterior and posterior positions. After screening, the dataset used in this experiment contains 286 SPECT images.

The thoracic region is a common site for bone metastases in lung cancer, so we cropped the image to 256 × 256 pixels using the method in [13], and only the thoracic region was retained in the cropped image.

#### Image labelling

SPECT image segmentation is a supervised task that requires accurate and reliable lesion segmentation annotation maps as the gold standard. To facilitate the labelling of lesions, this experiment used the SPECT image annotation system developed by our team based on LabelMe [14], an open-source online tool released by MIT. Three physicians with many years of clinical experience in nuclear medicine in the group manually labelled the areas of bone metastasis lesions, and the labelling information included custom symbols, disease names or body parts to ensure that the labelling information was correct. During the labeling process, each doctor annotates each image and discusses regions of inconsistency among the three annotators, and then votes to determine the final labeling result. After the annotation is completed, each image corresponds to a real segmentation map.

As shown in Table 1, in this experiment, we divided the dataset according to the ratio of training set: test set: 3, using 200 images as the training set and 86 as the test set. When dividing the dataset, we strictly placed the anterior and posterior images of the same patient into the same type of dataset because they will show similarity. There are 118 patients’ data contained in the training set and 53 patients’ data contained in the test set.

**Table 1:** Overview of the dataset used in this work

Parameter	Number of Images	Number of Patients
Training Set	200	118
Test Set	86	53
Total	286	171

### Overall architecture

In SPECT bone scan images, lung cancer lesions often exhibit a wide range of scale variation due to individual patient differences and the unpredictable distribution of cancer lesions. Additionally, other conditions such as inflammations, fractures, and residual radionuclide drugs in the spine may create regions of radionuclide hyperconcentration similar to those of metastatic bone lesions. These regions, with similar characteristics and wide-scale variations, present significant challenges for the accurate segmentation of lung cancer metastatic bone lesions. To address these issues, our proposed model focuses on enhancing multi-scale feature detection capabilities.

Specifically, we employ a generative adversarial network with an encoder-decoder architecture for the generator. Within the encoder, we integrate a cascade dilated convolution (CDC) module to enhance multi-scale feature extraction, while augmenting receptive fields through a multi-scale feature extraction (MSFE) module. In the decoder, we replace U-Net’s conventional convolution with a residual multi-scale (RMS) module [15], thereby expanding receptive fields without introducing additional parameters. To mitigate semantic loss from encoder downsampling, we employ an input image pyramid strategy. Additionally, we incorporate deep supervision and a multi-layer convolutional discriminator to refine segmentation, as proposed by Xue [16]. This approach enhances the model’s multi-scale segmentation capabilities via backpropagation.

**Fig. 1:**
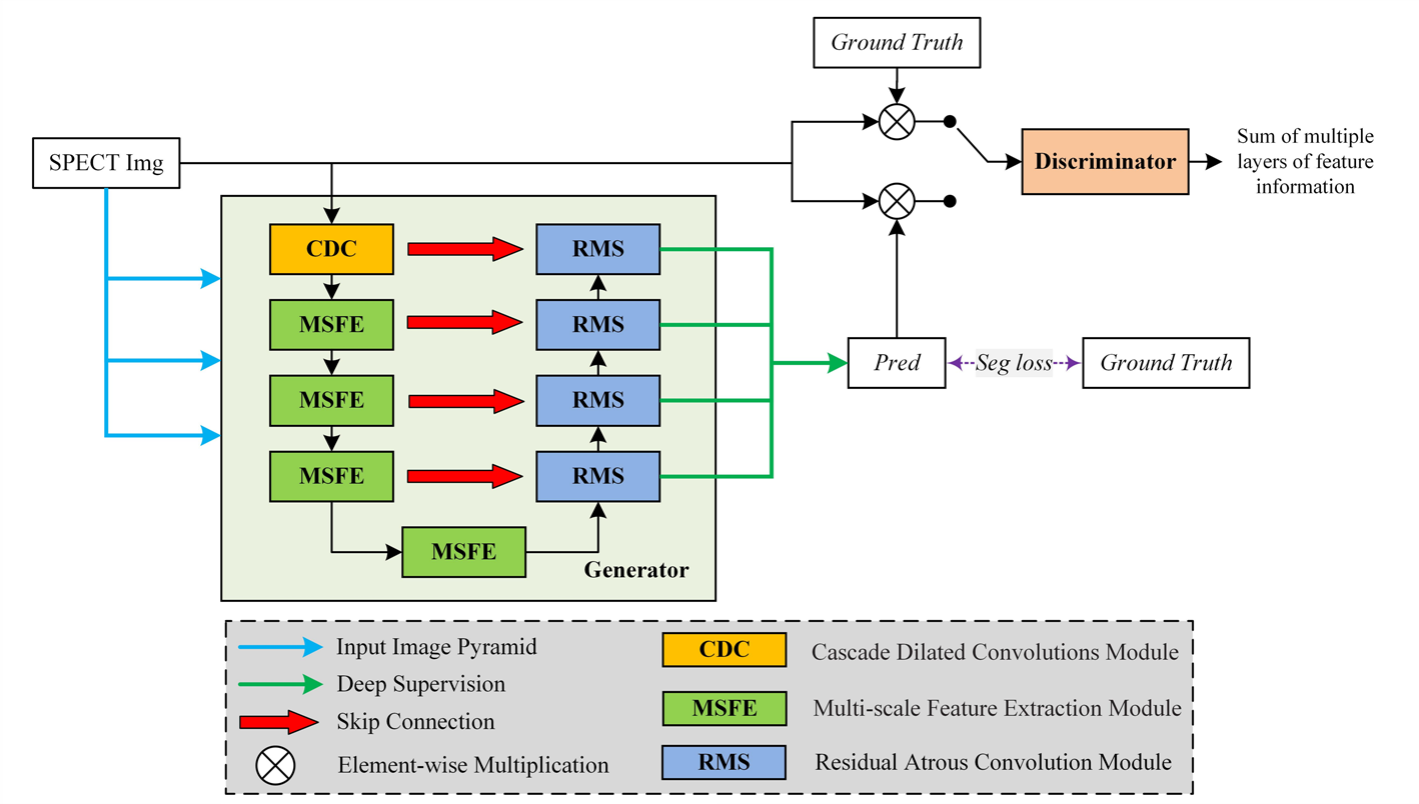
The workflow of the adversarial learning-based segmentation method for automatically identifying and delineating metastasis lesions in SPECT bone scintigrams. The generator integrates a MSFE module, CDC, and deep supervision to enhance sensitivity to lesions of varying scales, while the discriminator employs a multi-scale L1 loss computed over paired image–mask inputs to guide structure-aware learning

Figure 1 illustrates the workflow of the proposed segmentation method. The generator network generates a set of predictions based on the original image. These predictions are then combined with the original image and the labeled image (i.e., ground truth) using an element-wise multiplication operation. The combined image is subsequently fed into the discriminator network to determine its authenticity, resulting in a multi-scale L1 loss.

The generator and discriminator networks are elaborated below.

### The generator network

**Fig. 2:**
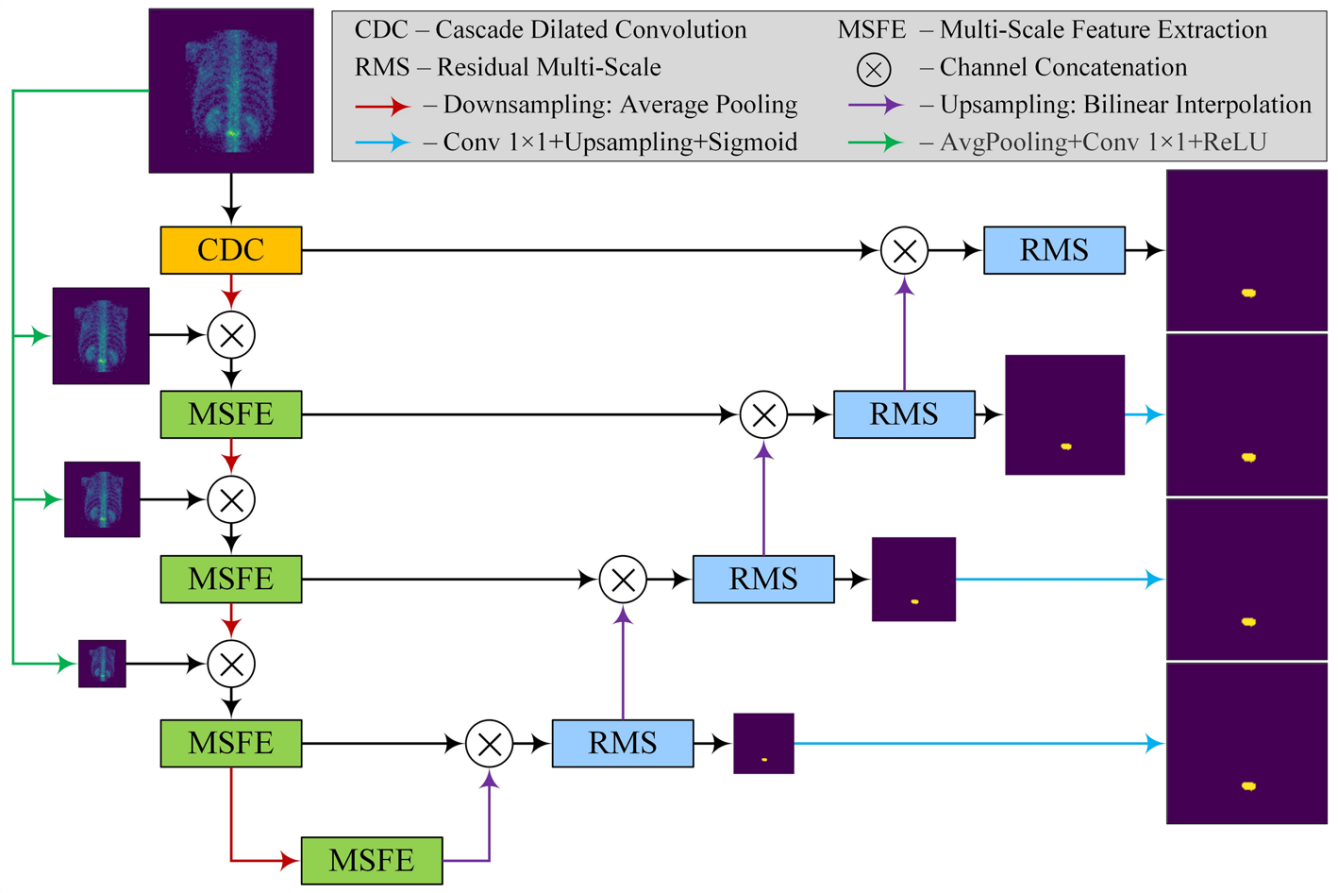
The structure of the proposed generator

To enable the model to focus on size-varied lesion objects in low-resolution bone scintigrams, we integrate three key techniques: the CDC module, MSFE module, and RMS module, to propose a lesion-sensitive generator. The structure of the generator network is depicted in Fig. 2.

As illustrated in Fig. 2, the proposed generator network follows the encoding-decoding architecture, where average pooling and bilinear interpolation are used for the encoding and decoding tasks, respectively.

#### Cascade dilated convolution (CDC)

**Fig. 3:**
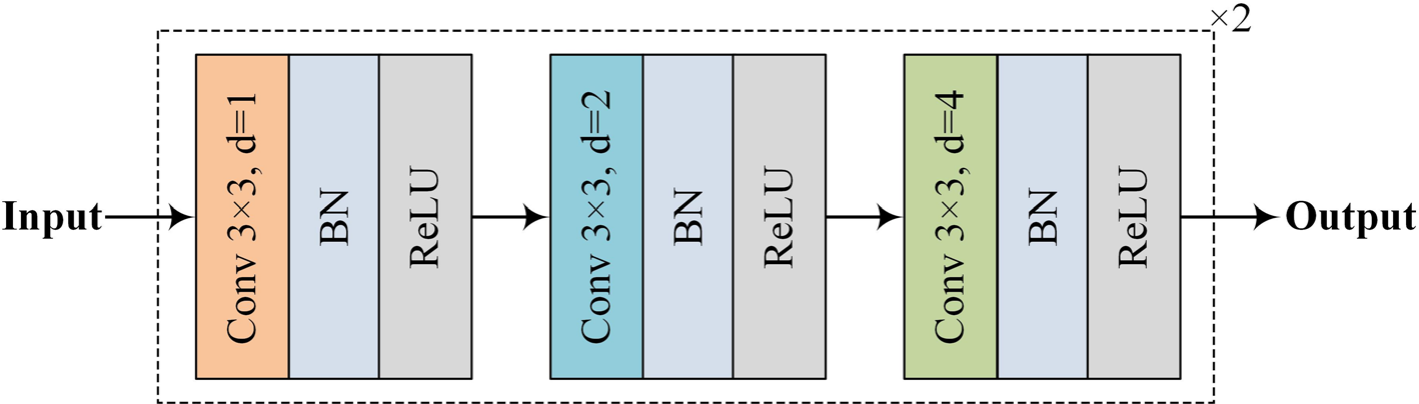
The structure of the CDC block used in the proposed generator network

As shown in Fig. 3, the original input image is first processed by a CDC block. Unlike the U-Net network, which uses a 3 × 3 convolution kernel, our CDC block employs three different dilation rates (i.e., d = 1, 2, 4) to capture the size-varied lesions. The duplicate configuration of CDC blocks allows the model to extract richer features of bone metastasis lesions.

The dilated convolutions used can effectively reduce the computational burden often associated with conventional convolutions using large kernels. The dilation rates of d = 1, 2, 4 are determined according to the design of hybrid dilated convolution (HDC) [17], which are robust enough to cope with the gridding effect.

After each dilated convolution, there is a batch normalization (BN) layer [18] and a ReLU function layer. Therefore, the reception fields for all six dilated convolution layers are 3, 7, 15, 17, 21, and 29 (see Table 2).

These values are calculated based on Eq. (1), and for clarity, we summarize them in Table 2 to provide an intuitive illustration of how different dilation rates affect the receptive field size across the CDC block.

This summary helps readers easily grasp the cumulative impact of dilation rates and their stacking order without the need for manual computation. The reception field, *rf* is calculated according to Eq. (1).

1 $$\:r{f}_{i}=\left\{\begin{array}{cc}1&\:i=1\\\:ks+\left(ks-1\right)\times\:\left(d-1\right)&\:i=2\\\:ks+\left(ks-1\right)\times\:\left(d-1\right)+s\times\:\left(r{f}_{i-1}-1\right)&\:i>2\end{array}\right.\:$$

**Table 2:** An illustration of calculating the reception fields with the input and its corresponding parameters in each dilated convolution layer

Input	Parameters	Reception field
Conv + BN + ReLU	*p* = 1, d = 1	3 × 3
	*p* = 2, d = 2	7 × 7
	*p* = 4, d = 4	15 × 15
	*p* = 1, d = 1	17 × 17
	*p* = 2, d = 2	21 × 21
	*p* = 4, d = 4	29 × 29

In Eq. (1) and Table 2, all convolutional layers are configured with fixed parameters: kernel size *ks* = 3 × 3, channels *c* = 64, stride *s* = 1. The variable parameters– padding *p* and dilation rate *d* −− are explicitly listed in Table 2.

#### Multi-scale feature extraction (MSFE)

**Fig. 4:**
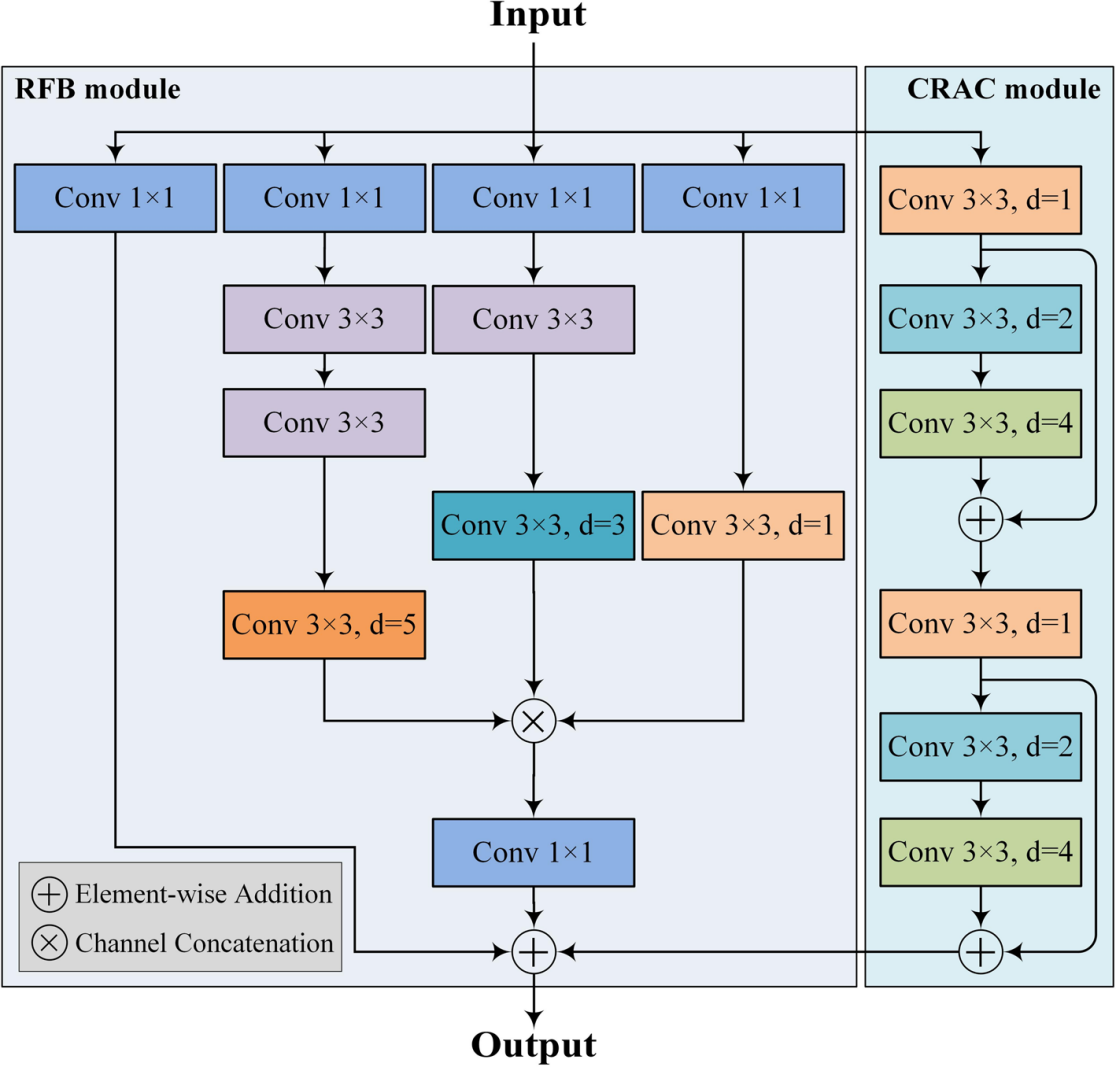
The structure of the MSFE block used in the proposed generator network. Each Conv module consists of a three-layer structure of Conv + BN + ReLU

The MSFE block is used from the 2^nd^ to 5^th^ layers in the generator network. An MSFE block consists of a cascade residual atrous convolution (CRAC) module and a reception field block (RFB) [19] module, depicted in Fig. 4. The “Conv” in the figure represents a convolutional module consisting of a “Conv-BN-ReLU” three-layer structure.

As shown in Fig. 4, a CRAC module is created by adding a residual connection path [20] to the CDC block to alleviate the vanishing gradient problem and accelerate convergence. Additionally, the RFB module of the MSFE block adopts the design of the Inception network [21]. This multiple-branch configuration helps the model to focus on lesions of different sizes.

Specifically, the CRAC module consists of six cascaded convolutional modules, where each convolutional module has a convolutional kernel size of 3 and expansion rates of 1,2,4,1,2,4, in that order. To alleviate the problem of gradient vanishing and accelerate the convergence of the model, residual connectivity is added between the first and the third, and the fourth and the sixth layers, respectively. The RFB module consists of a three-branched structure connected in parallel with a residual path. The equivalent convolutional kernel sizes of the three branches are, in order, 1,3,5. Convolutional layers with dilation rates of 1, 3, and 5 are added sequentially after the corresponding branches. Finally, the results from the three branches are summed element-wise with the residual paths and the CRAC module outputs, then processed by a ReLU activation function to obtain the final results of the MSFE module.

By integrating the multi-scale and dilated convolutions in parallel, our MSFE block can learn fine-grained features of the size-varied lesions from SPECT bone scintigrams. Using MSFE blocks in different layers enables the model to extract hierarchical features, including lower boundary and texture information, as well as higher semantic information.

#### Residual multi-scale module (RMS)

To enable the decoder to better predict lesion information at different scales, three cascaded dilated convolution-BN-ReLU structures [15] are used instead of the double-layer convolution in the U-Net model.

**Fig. 5:**
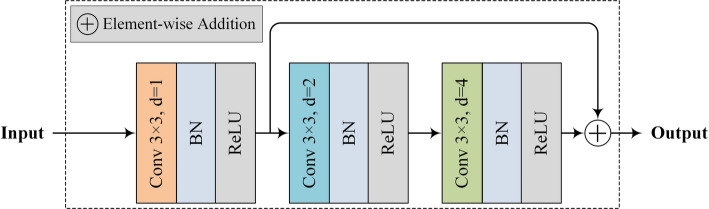
The structure of the RMS block used in the proposed generator network

As shown in Fig. 5, the dilation rates of the three dilated convolutions in the RMS module are 1,2, and 4, respectively, with residual connections added after the first and third layers. Unlike the two-layer convolution in the classical U-Net decoder, the dilated convolution expands the reception field without increasing the number of parameters. This enables the network to obtain more semantic information from lower resolution feature maps, reduces computational effort, and improves the model’s ability to accurately segment targets at different scales.

#### Input image pyramid

During the encoding stage, the repeated use of down-sampling and convolutions may result in information loss. This is problematic for the SPECT scintigrams with very low spatial resolution. To cope with this issue, an input image is halved using an averaging pooling operation at each layer. This reduced image is then concatenated with the output feature map of the previous layer (i.e., channel concatenation) to compensate for possible information loss.

Specifically, three images with sizes of 1/2, 1/4, and 1/8 of the original image are concatenated after the first, second, and third layers, respectively, as shown in Fig. 2 to provide compensation for feature maps.

#### Deep supervision

A typical observation is that a deeper network offers better performance but can lead to the vanishing gradient problem during model training. To address this, a deep supervision mechanism [22] is used to supervise each layer of the feature reconstruction process, providing feedback through a loss function to optimize training.

During the decoding stage, except for the last layer, a 1 × 1 convolution is used to reduce the number of channels to 1 in each layer. Bilinear interpolation is then utilized to rescale the feature map to the same size as the input. Finally, a sigmoid function is applied in each layer to limit the pixel value to a range of 0 to 1. During the training process, the four predicted segmentation maps output by the segmentation network are compared with the real segmentation maps to compute the adversarial loss and segmentation loss, respectively. These losses are then summed to obtain the overall adversarial loss and segmentation loss.

With the deep supervision strategy, supervisory signals are added to different layers of the segmentation network decoder, allowing feedback and gradient updates at various stages. By making predictions at multiple layers, the network can utilize both local and global information, thus improving the ability to perceive structures at different scales and enabling the model to produce robust segmentation results for targets of varying sizes.

### The discriminator network

Unlike traditional generative adversarial networks (GANs), in this work, we use the authenticity label map and multiply it by the corresponding elements of the original map to obtain the original map mask. This mask is then fed into the discriminator, and the L1 loss between the two is calculated as the adversarial loss for both the generator and the discriminator.

**Fig. 6:**
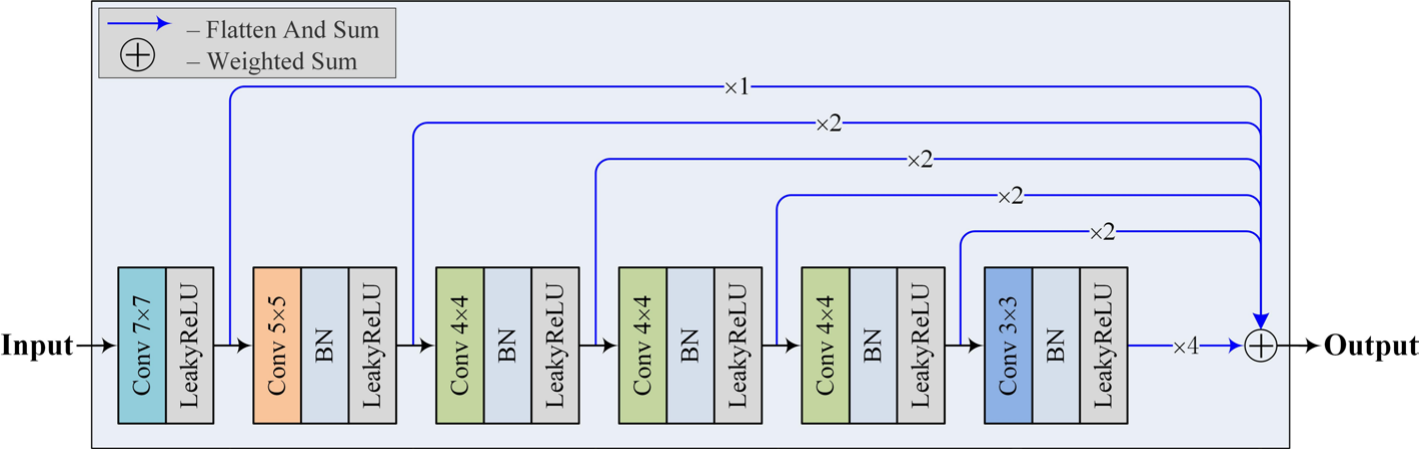
The structure of the discriminator

We use the discriminator network proposed in [16], as shown in Fig. 6. The discriminator extracts different levels of features through a multilayer convolutional structure, then flattens and weights them into a one-dimensional tensor to obtain the final result. During training, the original image masks corresponding to the predicted and real segmentation results are fed into the discriminator to obtain the respective sums, and then the L1 loss between them is calculated as the adversarial loss. By fusing features from different levels, the discriminator can capture the long and short distance spatial relationships between pixels at multiple scales, improving the perception of lesions at different scales.

In the traditional GAN network, the discriminator outputs the probability that the input label is true. However, in our dataset, experiments show that this scheme makes the discriminator too strong to form the correct training feedback to the generator, leading to an unstable or even failed training process. Moreover, simple one-dimensional probability values cannot provide stable and sufficient gradient feedback to the network. Therefore, in the discriminator, we choose to output the weighted sum of the output results of the input data at different convolutional layers, thus obtaining the overall representation of the original image mask at different scales. On the SPECT image, the multi-scale L1 loss achieves better segmentation results.

### Loss function

For the adversarial learning-based segmentation framework, the overall objective is to maximize the generation capability of the generator *G*, while minimizing the discriminative ability of the discriminator *D*. The joint optimization objective is expressed as:

2 $$\:\zeta\:\left(G,D\right)=L\left(G\right)+L\left(D\right)\:$$

Here, *L*(*G*) represents the generator loss, and *L*(*D*) denotes the discriminator loss. The detailed formulation of each component is given below.

1. **Generator Loss** ***L(G)***

The generator loss *L*(*G*) comprises two components: a segmentation loss *L*_*seg*_*(G*) and an adversarial loss *L*_*adv*_(*G*):

3 $$\:L\left(G\right)={\alpha\:\:L}_{seg}\left(G\right)+{\beta\:\:L}_{adv}\left(G\right)\:$$

where *α* and *β* are weighting coefficients to balance the two terms.

#### Segmentation loss

To effectively guide the segmentation network, we adopt the Dice loss, which is widely used in medical image segmentation. The segmentation loss is averaged over four decoding layers and defined as:

4 $$\:{L}_{seg}\left(G\right)=\frac{1}{4}{\sum_{i=1}^{4}}\left(1-Dice\left({y}_{i},gt\right)\right)\:$$

where *y*_*i*_ denotes the segmentation prediction at the *i*-th layer, and *gt* is the corresponding ground truth.

#### Adversarial loss

The adversarial component encourages the generated segmentation to be indistinguishable from the ground truth when fused with the input image. It is defined as.

5 $$\:{L}_{adv}\left(G\right)=\frac{1}{4}{\sum_{i=1}^{4}|D}\left({y}_{i}\times\:image\right)-D\left(gt\times\:image\right)|\:$$

(2) **Discriminator Loss** ***L(D)***

The discriminator aims to distinguish between real (ground truth) and fake (generated) segmentation masks. Its loss is formulated as:

6 $$\:L\left(D\right)={L}_{adv}\left(D\right)=1-\frac{1}{4}{\sum_{i=1}^{4}|D}\left({y}_{i}\times\:image\right)-D\left(gt\times\:image\right)|\:$$

### Experimental setup

We trained and tested the model using a PC with a 15 vCPU AMD EPYC 7543 32-core processor and A40 (48GB) GPU to meet the model’s computational power requirements. The experiments were implemented and executed using the PyTorch 1.13.0 framework. The model training parameters are shown in Table 3.

**Table 3:** Parameters of the proposed model

Parameter	Value
Learning Rate	0.0001
Optimizer	Adam
Batch Size	32
Epoch	600

When generating predictive labels, a threshold of 0.5 was uniformly specified, where pixels greater than or equal to 0.5 were categorized as the foreground of the lesion and pixels less than 0.5 were categorized as the background.

The trained model is run 5 times on the training subset to minimize the effect of randomness. For each metric defined above, the final output of the model is the average of the results of the 5 runs. The experimental results reported in the following sections are averages unless otherwise stated. For this experiment, we set the value of the random seed to 42.

### Evaluation metrics

This experiment uses Dice Similarity Coefficient (DSC), precision and recall as evaluation metrics. The definitions are given in Eqs. (7)–(9).

7 $$\:DSC=\frac{2\times\:Precision\times\:Recall}{Precision+Recall}\:$$

8 $$\:Precision=\frac{TP}{TP+FP}\:$$

9 $$\:Recall=\frac{TP}{TP+FN}\:$$

where *TP* denotes true positive, *TN* denotes true negative, *FP* denotes false positive and *FN* denotes false negative. In this paper, the evaluation metrics are reported as the mean ± standard deviation.

## Results

### The proposed model performance

For the segmentation model we proposed, Table 4 presents the evaluation metrics scores obtained by the generator on the test samples of the dataset. The results are reported as mean ± absolute standard deviation: DSC = 0.6671, Precision = 0.7228, Recall = 0.6196.

**Table 4:** Evaluation index scores of the proposed model on the test set. The values represent means ± absolute standard deviations

Evaluation metrics	DSC	Precision	Recall
**Value**	0.6671 ± 0.0027	0.7228 ± 0.0145	0.6196 ± 0.0114

### Comparison experiment

This section compares the proposed models with classical and adversarial learning-based segmentation models using consistent training parameters. Table 5 presents the evaluation metrics for each model on the test samples, reported as mean ± absolute standard deviation.

From the quantitative perspective, it can be seen that our proposed model obtained the highest DSC and recall metric scores on the test samples. This indicates that the model demonstrates greater superiority and feasibility in segmenting metastatic bone lesions in SPECT images. In addition, although U-Net+++ has better results in precision metrics, the overall U-Net+++ segmentation performance is lower than that of our proposed model. The possible reasons are that on the one hand, through adversarial learning, our model can better learn the feature information of lesions better; on the other hand, our designed cascaded null convolution module and multi-scale feature extraction module enriches the model’s sensory field, which enhances the model’s ability to detect and segment multi-scale lesions.

**Table 5:** Comparison of segmentation results of the proposed model with the classical model. Errors are reported as absolute standard deviations

Model	DSC	Precision	Recall	*p*(DSC)	*p*(Precision)	*p*(Recall)
U-Net [3]	0.6311 ± 0.0100	0.7221 ± 0.0416	0.5628 ± 0.0309	< 0.0001	0.9309	< 0.0001
Attention U-Net [23]	0.6314 ± 0.0126	0.7541 ± 0.0257	0.5430 ± 0.0218	< 0.0001	< 0.0001	< 0.0001
U-Net++ [24]	0.6228 ± 0.0052	0.7291 ± 0.0313	0.5436 ± 0.0371	< 0.0001	0.3213	< 0.0001
U-Net+++ [25]	0.6470 ± 0.0034	**0.7565 ± 0.0168**	0.5652 ± 0.0279	< 0.0001	< 0.0001	< 0.0001
SegNet [1]	0.6345 ± 0.0274	0.6725 ± 0.0372	0.6006 ± 0.0190	< 0.0001	< 0.0001	< 0.0001
DoubleUNet [26]	0.6455 ± 0.0058	0.7485 ± 0.0284	0.5675 ± 0.0187	< 0.0001	0.2619	< 0.0001
CE-Net [27]	0.6335 ± 0.0136	0.6856 ± 0.0157	0.5889 ± 0.0392	< 0.0001	< 0.0001	< 0.0001
PSPNet [28]	0.4556 ± 0.0326	0.5335 ± 0.0351	0.3976 ± 0.0348	< 0.0001	< 0.0001	0.0153
Liu et al. [10]	0.6506 ± 0.0137	0.6535 ± 0.0279	0.6477 ± 0.0320	< 0.0001	< 0.0001	0.2714
Khan et al. [11]	0.6522 ± 0.0388	0.6899 ± 0.0317	0.6184 ± 0.0271	< 0.0001	< 0.0001	0.0024
Innani et al. [12]	0.6463 ± 0.0263	0.6985 ± 0.0292	0.6014 ± 0.0210	< 0.0001	< 0.0001	0.0024
nnU-Net [29]	0.6583 ± 0.0326	0.7325 ± 0.0423	0.6128 ± 0.0221	< 0.0001	< 0.0001	**0.0017**
***Proposed***	**0.6671 ± 0.0027**	0.7228 ± 0.0145	**0.6196 ± 0.0114**	**—**	**—**	**—**

To further assess whether the observed performance improvements of the proposed method are statistically significant, we conducted two-sample t-tests (*n* = 30) comparing the proposed method with each baseline model on the DSC, Precision, and Recall metrics. The *p*-values from these tests are summarized in Table 5. Results show that the proposed method achieves statistically significant improvements (*p* < 0.05) in DSC and Recall compared with all baselines, while Precision differences are less consistent.

The proposed model (57 M parameters) exhibits higher complexity and resource demands than the nnU-Net baseline (45 M parameters). Specifically, it requires 19 GB of GPU memory during training (vs. 14 GB) and has an inference time of 1.5 s per sample (vs. 1.2 s). This increased computational burden represents a necessary trade-off for the novel architectural modifications and adversarial learning mechanisms employed. As demonstrated in Table 5, these enhancements yield significantly superior performance on SPECT image tasks.

**Fig. 7:**
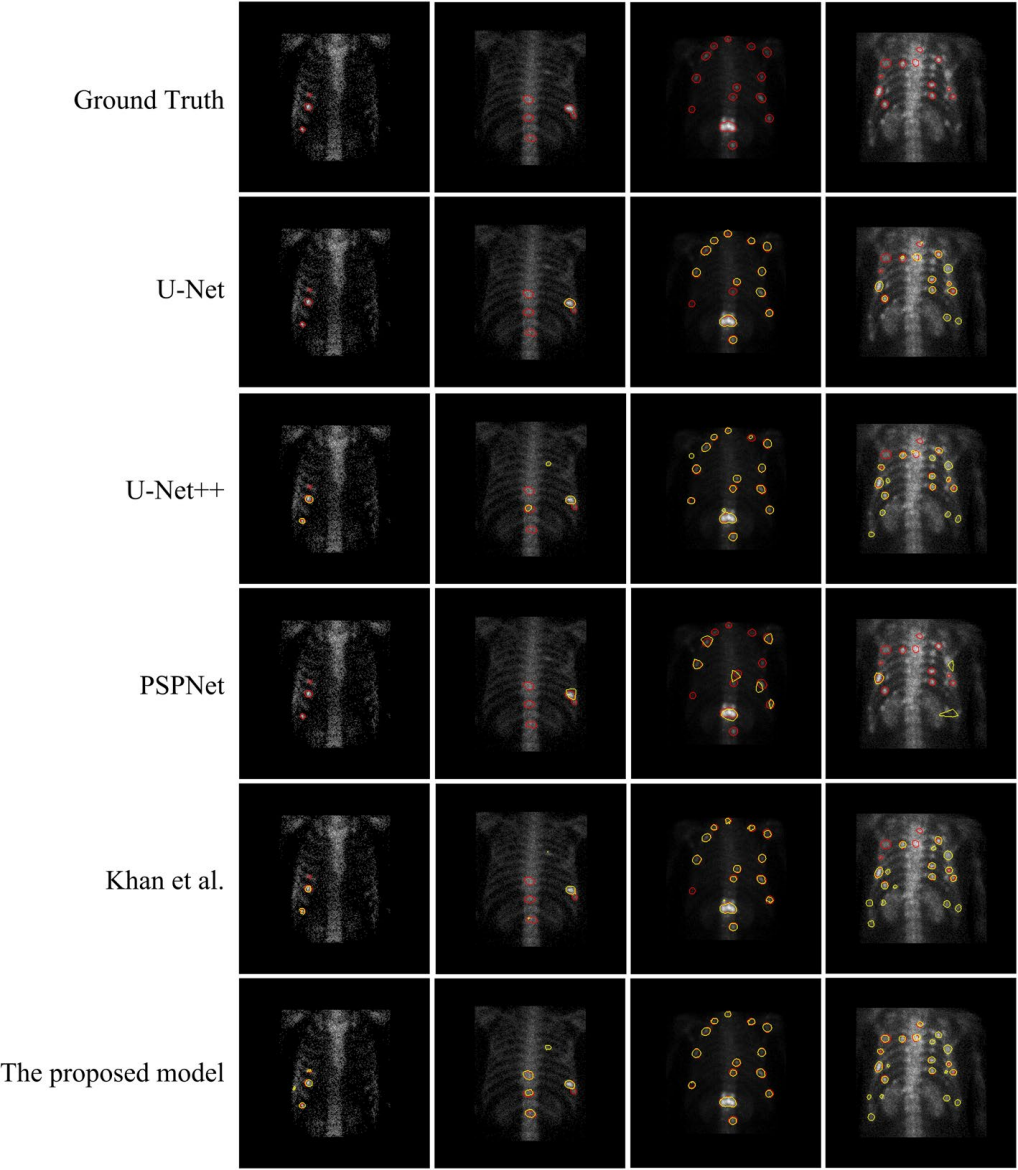
Segmentation effect of various medical image segmentation models and our proposed model on test samples

A qualitative comparison between our proposed model and the classical medical image segmentation model in terms of segmentation results is presented in Fig. 7, where the region enclosed by the red curve represents the predicted segmentation result and the region enclosed by the yellow curve depicts the real segmentation result.

It can be seen that PSPNet produces the worst segmentation results; it predicts only a small number of large-scale lesions, and the edges of the predicted lesions are so rough that they cannot be applied to the task of SPECT image segmentation. For small-scale lesions in areas such as the clavicle and the end of the ribs, their small area and large similarity in features, such as size and shape, to the background make it difficult for the classical segmentation model to segment them accurately. On the contrary, our proposed model shows better segmentation performance for small-scale lesions by designing receptive fields of different sizes.

The generally high pixel value of the spine region in SPECT images makes the hot area of the lesion easily confused with the spine background and results in the transition blurring of the boundary region of the lesion, posing a challenge to the accurate segmentation of the model. It can be seen that the classical segmentation model is not very effective in segmenting some of the spinal lesions, and suffers from underreporting and inaccurate lesion edge prediction. On the contrary, our proposed model shows better segmentation performance in the spine part due to its rich receptive field and therefore better learning of the spatial relationship between long and short distances between pixels.

In addition, for the case of multiple lesions clustered together, neither our model nor the classical segmentation model can effectively segment all lesions clustered together, which inspires us to focus more on the case of multiple lesions clustered together to develop better segmentation models in our future work.

### Ablation experiment

In the ablation experiments, we used the RMS segmentation model as the baseline model and sequentially added the adversarial network, the input image pyramid, the deep supervision, the MSFE module, and the CDC module to test their respective effects on model performance. Table 6 demonstrates the evaluation metric scores of different improvement components on the test samples.

**Table 6:** Ablation experiment results

RMS	GAN	Pyramid^1^	DeepSpv^2^	MSFE	CDC	DSC	Precision	Recall
✓	×	×	×	×	×	0.6487 ± 0.0054	0.7233 ± 0.0335	0.5880 ± 0.0227
✓	✓	×	×	×	×	0.6511 ± 0.0030	**0.7280 ± 0.0155**	0.5889 ± 0.0918
✓	✓	✓	×	×	×	0.6530 ± 0.0034	0.7167 ± 0.0333	0.5998 ± 0.0295
✓	✓	✓	✓	×	×	0.6536 ± 0.0032	0.7005 ± 0.0268	0.6126 ± 0.0275
✓	✓	✓	✓	✓	×	0.6640 ± 0.0031	0.7241 ± 0.0208	0.6131 ± 0.0322
✓	✓	✓	✓	×	✓	0.6573 ± 0.0029	0.7040 ± 0.0146	0.6164 ± 0.0287
✓	✓	✓	✓	✓	✓	**0.6671 ± 0.0027**	0.7228 ± 0.0145	**0.6196 ± 0.0114**

^1^ Input Image Pyramid; ^2^ Deep Supervision

Table 6 shows that the DSC, precision and recall scores of the benchmark RMS model on the test set are 64.86%, 72.33% and 58.80% respectively. Compared to Table 5, the benchmark model achieves higher evaluation metric scores than the classical segmentation model. We added the discriminator and adversarial training part to the benchmark RMS model to provide additional adversarial loss to the segmentation network. This resulted in significant improvements in the DSC and precision metrics, 0.24% and 0.46%, respectively, and provides a slight improvement in the recall metric, 0.09%.

The addition of the input image pyramid and deep supervision further improves the model in terms of DSC and recall metrics. We then add the MSFE module and the CDC module to the model, respectively, to reflect the contribution of both to the model. The MSFE module improves the DCS, precision and recall metrics by 1.03%, 2.36% and 0.04% respectively, while the CDC module improves the DCS, precision and recall metrics by 0.37%, 0.35% and 0.38%, respectively, compared to the previous configuration. Compared to the CDC module, the MSFE module improves the model more significantly on the DSC and precision metrics, indicating that our proposed multi-scale feature extraction module can effectively enhance the model’s ability to learn lesion features at different scales, thus providing more complete and accurate semantic information for the generator-decoder paths. The CDC module contributes more to the model in recall metrics, which may be because the CDC module is located at the shallowest level of the segmentation network encoder, providing more details, edges and texture information for the generator to generate the final segmentation labels.

Compared to the benchmark model, our proposed model improves 1.84% and 3.16% in DSC and recall metrics, respectively, and is basically unchanged in precision metrics. From the perspective of medical clinical practice, the lower leakage rate of the model with higher segmentation accuracy can help physicians to reduce the leakage of difficult-to-discriminate types of lesions, thus better avoiding the delay of patient treatment due to undetected lesions.

#### Segmentation loss comparison

Table 7 shows the model performance with different loss function combinations employed by the generator. As shown in the table, the model performs the worst when the generator uses BCE Loss, with DSC decreasing by 2.62% and recall decreasing by 5.33% compared to the best case. Compared to Dice Loss + BCE Loss, the model using only Dice Loss, although lower in precision metrics, shows a significant advantage in DSC and recall. This is mainly because BCE Loss treats each pixel as a sample independent of each other and does not take into account the spatial relationship between pixels, which makes it difficult for the model to deal with the blurring of lesion edges. Additionally, the lesion edge transition in SPECT images is not obvious, and the image resolution is low, which further highlights the defects of BCE Loss itself. Therefore, the combination of Dice Loss + BCE Loss used in the model is inferior to Dice Loss in terms of metrics.

**Table 7:** Performance of the generator with different loss functions

Dice Loss	BCE Loss	DSC	Precision	Recall
✓	×	**0.6671 ± 0.0027**	0.7228 ± 0.0145	**0.6196 ± 0.0114**
×	✓	0.6409 ± 0.0082	0.7381 ± 0.0241	0.5663 ± 0.0154
✓	✓	0.6581 ± 0.013	**0.7496 ± 0.0215**	0.5865 ± 0.0269

#### CRAC expansion rate comparison

In order to investigate the effect of different sensory fields on the segmentation performance of the model, we tried using different combinations of expansion rates for the six cascade convolutional layers in the CRAC module of the MSFE module and obtained the results in Table 8. In the table, 3# is the expansion rate used in our proposed model. For example, 1# represents the expansion rate of the CRAC module from input to output for its six cascade convolutional layers in the order of 1,2,3,1,2,3.

As can be seen from Table 8, the model achieved the best segmentation results when the expansion rate of the cascaded convolutional layers was the 3# case. When gradually decreasing or increasing the expansion rate, i.e., changing the modular receptive field, the segmentation performance of the model significantly decreased. This indicates that our proposed CRAC modular receptive field design is advantageous for lesion segmentation in SPECT images. When the receptive field is too small, the model tends to neglect the detection of large-scale lesions, while when the receptive field is too large, it negatively affects the segmentation of small-scale lesions.

**Table 8:** Experimental results of applying different expansion rates in CRAC

Case	Dilation Rates	DSC	Precision	Recall
Case #1	1,1,1,1,1,1	0.6512 ± 0.0023	0.7047 ± 0.0159	0.6052 ± 0.0245
Case #2	1,2,3,1,2,3	0.6603 ± 0.0034	0.7171 ± 0.0245	0.6118 ± 0.0142
Case #3 (proposed)	1,2,4,1,2,4	**0.6671 ± 0.0027**	0.7228 ± 0.0145	**0.6196 ± 0.0114**
Case #4	1,3,5,1,3,5	0.6608 ± 0.0057	**0.7327 ± 0.0218**	0.6017 ± 0.0316
Case #5	1,5,7,1,5,7	0.6653 ± 0.0136	0.7215 ± 0.0361	0.6172 ± 0.0247

#### Impact of decoder layer weights on segmentation performance

To examine how segmentation performance varies with the importance of different layers in the segmentation network, we applied various weighting schemes to the loss functions of the four outputs along the decoder path. Table 9 presents the segmentation metrics under different weighting settings, where D1 represents the deepest output and D4 denotes the shallowest output of the decoder, i.e., the final segmentation output.

Table 9 reveals that the model performs best when equal weights are applied to the loss functions of all four decoder layers. Increasing the weights of the shallow layers results in a decrease in DSC but an increase in recall, while weighting the deep and intermediate layers does not enhance performance. This suggests that assigning higher weights to the shallow layers allows the model to better learn shallow features, such as lesion edges and textures.

**Table 9:** Generator decoder weight change results for different layers of the corresponding loss

Case	D1	D2	D3	D4	DSC	Precision	Recall
Case #1 (proposed)	1	1	1	1	**0.6671 ± 0.0027**	0.7228 ± 0.0145	0.6196 ± 0.0114
Case #2	0.4	0.4	0.1	0.1	0.6595 ± 0.0064	0.7216 ± 0.0195	0.6073 ± 0.0245
Case #3	0.1	0.1	0.4	0.4	0.6631 ± 0.0057	0.6887 ± 0.0205	**0.6394 ± 0.0216**
Case #4	0.1	0.4	0.4	0.1	0.6593 ± 0.0175	**0.7275 ± 0.0229**	0.6028 ± 0.0282

#### Effect of loss function weight coefficients

To precisely determine the weight values in Eq. 5 and evaluate their effects on the model, we tested various combinations of the two parameters. The results are presented in Table 10. In this study, the final weight values for α and β were set to 1 and 1, respectively.

Table 10 evaluates five weight combinations, with the (1,1) coefficient proving to be the most effective. These results indicate that segmentation loss and adversarial loss contribute equally to the segmentation of bone metastatic lesions in SPECT images.

**Table 10:** The hyperparameter experimental results illustrate the effect of *α* and *β* parameters

α	β	DSC	Precision	Recall
1	1	**0.6671 ± 0.0027**	0.7228 ± 0.0145	**0.6196 ± 0.0114**
0.1	0.9	0.6434 ± 0.0136	**0.7421 ± 0.0238**	0.5679 ± 0.0322
0.3	0.7	0.6540 ± 0.0121	0.7292 ± 0.0257	0.5927 ± 0.0240
0.7	0.3	0.6550 ± 0.0078	0.7016 ± 0.0233	0.6142 ± 0.0351
0.9	0.1	0.6570 ± 0.0031	0.7096 ± 0.0188	0.6116 ± 0.0205

#### Effect of inputting discriminator data

Unlike typical conditional GANs, we use a mask created by multiplying the segmentation labels with the original image, which is then fed into the discriminator. To assess the effectiveness of this approach, we tested various combinations of the original image and segmentation labels, as detailed in Table 11.

Table 11 shows that using the original image mask yields the best segmentation performance, while using only the input label results in the poorest performance. Both splicing and summing the original image with the label also perform worse than the image mask. This suggests that for SPECT bone metastasis lesion segmentation, channel splicing and pixel summation do not provide sufficient feature information for the adversarial network.

**Table 11:** The experimental results illustrate the effect of different input data on the discriminator

Data format	DSC	Precision	Recall
image * pred/gt	**0.6671 ± 0.0027**	**0.7228 ± 0.0145**	**0.6196 ± 0.0114**
image concat pred/gt	0.6520 ± 0.0517	0.6913 ± 0.0157	0.6175 ± 0.0172
image + pred/gt	0.6520 ± 0.0035	0.6979 ± 0.0103	0.6120 ± 0.0110
pred/gt	0.6424 ± 0.0031	0.6866 ± 0.0409	0.6064 ± 0.0314

## Discussion

This paper presents an adversarial learning-based segmentation model for lung cancer bone metastasis in SPECT images. The model improves segmentation accuracy by incorporating multiscale L1 loss, CDC, and MSFE modules, which enhance the generator’s ability to manage varying lesion sizes and shapes. Tested on 286 SPECT images, the proposed model outperforms existing approaches in segmenting lesions across different scales.

**Fig. 8:**
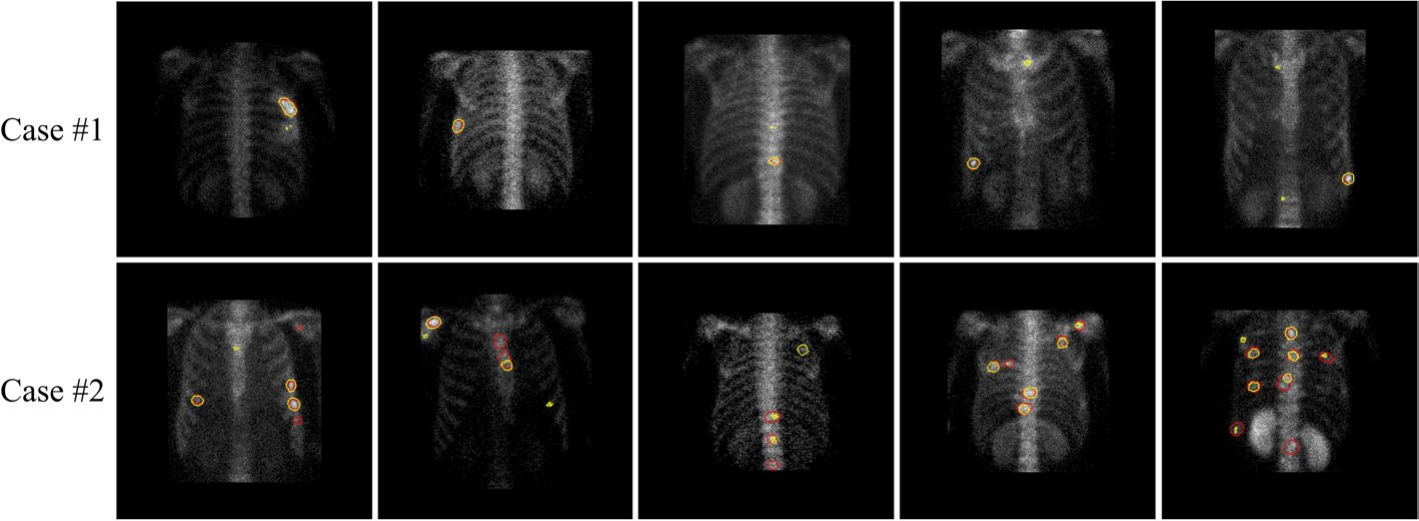
Segmentation effect of our proposed model on single lesion images and multi-lesion images

However, the model has limitations. As shown in Fig. 8, it performs better on single-lesion images (Case #1) compared to multi-lesion images (Case #2), with the edges of lesions nearly perfectly aligned with the ground truth. In contrast, the model struggles with false positives (*FP*) and false negatives (*FN*) in multi-lesion images. This may be due to the high sensitivity and low specificity of SPECT images, coupled with the variability in size and shape of metastatic bone lesions compared to natural images. This variability can lead to confusion with the background or benign areas, complicating accurate segmentation and resulting in false positives and omissions.

Several factors negatively impact segmentation performance.

**Fig. 9:**
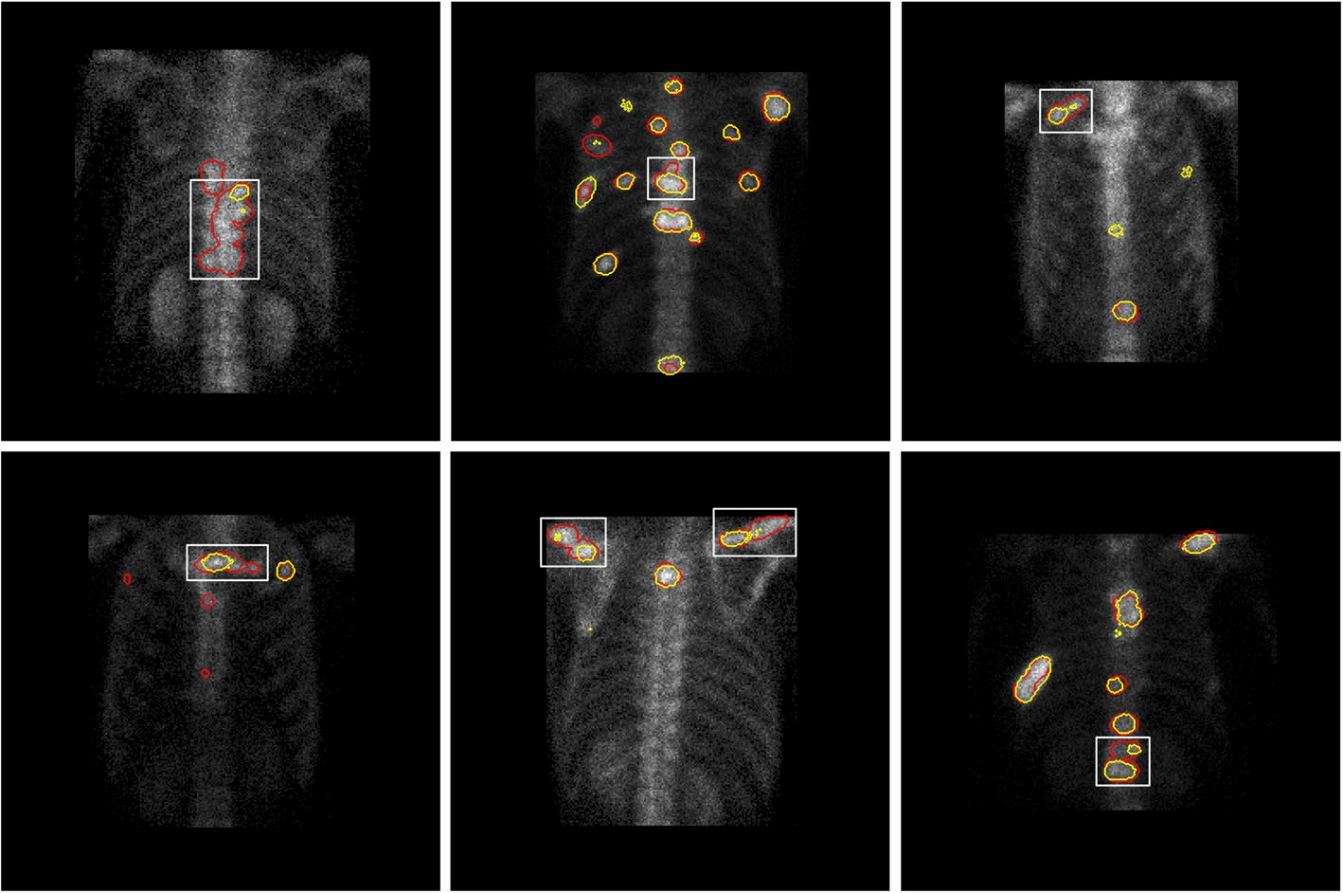
Segmentation results of the proposed model on a case with multiple lesion aggregations. The yellow contour represents the ground truth segmentation, while the red contour indicates the predicted segmentation

The aggregation of multiple lesions often leads to irregular shapes. In our dataset, most lesions appear individually as circular or oval shapes. Our model is optimized to predict such shapes. However, due to varying patient conditions, some lesions cluster together, causing edge overlaps and creating large, irregular areas in the segmentation labels. This clustering is less common in the dataset, so the model does not learn to handle it effectively, resulting in false negatives. The clustering of multiple lesions is highlighted in the white box in Fig. 9. The model attempts to segment this aggregated region as separate small lesions, focusing on their central parts. While somewhat effective, this approach fails to accurately capture the edge regions of the lesions.

Benign bone lesions (e.g., fractures, arthritis) and areas with high nuclide uptake (e.g., spine) in SPECT images also form hot zones similar to metastatic lesions. Additionally, nuclide uptake varies between patients due to metabolic differences, leading to significant variations in lesion size, shape, and intensity. These variations result in false positives and complicate the model’s ability to learn and segment metastatic lesions accurately. Dice loss, being pixel-wise, does not account for the number of detected or missed lesions, which poses a challenge in medical image segmentation.

Constructing large-scale datasets from a greater number of patient SPECT images is challenging due to difficulties in collecting real patient images, owing to privacy concerns and the rarity of the disease. Small-scale datasets lead to inadequate training and lower segmentation performance for convolutional neural network-based models. Therefore, we need to create large-scale SPECT image datasets, with manual annotation and verification by professional physicians, to enhance the performance of the segmentation model.

Although the average performance improvement of our method over other state-of-the-art models appears modest (e.g., + 2.28% in DSC), we contend that this gain is clinically meaningful given the inherent variability and complexity of SPECT imaging. Notably, our model achieves the lowest standard deviation (± 0.0027 in DSC), suggesting greater prediction consistency across diverse patient cases.

Furthermore, when comparing the best-case performance of our model (mean + standard deviation) to the worst-case performance (mean − standard deviation) of prior adversarial approaches such as Innani et al. [12], we observe substantial relative improvements: +58.35% in DSC, + 47.93% in Precision, and + 73.93% in Recall. These margins underscore the upper-bound potential of our proposed framework.

It is also worth noting that in the adversarial segmentation literature, performance gains are typically incremental (< 3%). Our integration of multi-scale modules and structure-aware loss helps surpass this threshold, especially for small and difficult lesions.

In future work, we will explore domain-adapted nnU-Net configurations to more rigorously validate our approach under broader benchmarking standards. We also plan to perform complexity and resource analysis to assess the practical deployability of the proposed method.

To substantiate the observed improvements, we have conducted statistical significance testing (Table 8) and a comprehensive ablation study (Table 6). These experiments demonstrate that each module, such as CDC, MSFE, Pyramid, and DeepSupervision, provides consistent, quantifiable improvements. This modular transparency ensures that the model’s performance gains are not incidental or a result of overfitting.

In summary, the above discussion outlines both the strengths and limitations of the proposed model in handling complex lesion patterns in SPECT imaging, highlighting its robustness, generalizability, and clinical relevance.

## Conclusions

This paper presents an adversarial learning-based segmentation model for lung cancer bone metastasis lesions in SPECT images. Our approach introduces the adversarial learning concept into the image segmentation task to improve the higher-order consistency between the generated labels and real labels by computing the multiscale L1 loss of the image mask. The generator network is enhanced by replacing the original double-layer 3 × 3 convolution with the CDC module and MSFE module, combining the input image pyramid and depth supervision techniques to enrich the model’s range of sensory fields, thus effectively improving the generator’s multiscale segmentation capability. The proposed MSFE module has demonstrated better identification of metastatic bone lesions of lung cancer with varying size, shape and location, significantly improving the prediction of lesion boundaries. Experiments were conducted on a dataset consisting of 286 SPECT images to segment metastatic bone lesions of lung cancer. The experimental results indicate that the proposed model is more effective in accurately segmenting lesions at different scales compared to existing classical medical image segmentation models.

While the DSC score of our model (0.6671) falls slightly below the commonly accepted threshold for fully automated clinical deployment, it remains well-suited for pre-screening, triage, and decision support applications, especially in high-workload environments or resource-limited radiology settings. The model’s enhanced recall and low prediction variance further support its utility as an assistive diagnostic tool, aiding clinicians in detecting subtle or clustered metastatic lesions that may otherwise go undetected.

In the future, we plan to expand our work in the following directions. First, we aim to collect more SPECT lung cancer bone metastasis data to further train the model. Second, we will conduct further research into the clustering of lung cancer bone metastases and attempt to develop a bone metastasis lesion segmentation model for the case of multiple lesion clustering. Finally, we intend to develop a computer-aided diagnosis system based on the model, which would assist physicians in clinical applications.

## Data Availability

Anyone can get the validation subset by emailing the corresponding author by stating that the data is used for research purposes only. The whole dataset will be publicly available in the future.
